# Multicentre study on the reproducibility of MALDI-TOF MS for nontuberculous mycobacteria identification

**DOI:** 10.1038/s41598-022-05315-7

**Published:** 2022-01-24

**Authors:** David Rodriguez-Temporal, Fernando Alcaide, Ivana Mareković, James Anthony O’Connor, Rebecca Gorton, Jakko van Ingen, An Van den Bossche, Genevieve Héry-Arnaud, Clémence Beauruelle, Dorothea Orth-Höller, Juan-José Palacios-Gutiérrez, Griselda Tudó, Germán Bou, Pieter-Jan Ceyssens, Montserrat Garrigó, Julià González-Martin, Gilbert Greub, Jaroslav Hrabak, André Ingebretsen, Maria Concepción Mediavilla-Gradolph, Marina Oviaño, Begoña Palop, Arthur B. Pranada, Lidia Quiroga, Maria Jesús Ruiz-Serrano, Belén Rodríguez-Sánchez

**Affiliations:** 1 Department of Clinical Microbiology and Infectious Diseases, Hospital General Universitario Gregorio Marañón, Madrid, Spain; 2 Servei de Microbiologia, Hospital Universitari de Bellvitge-IDIBELL, Departament de Patologia i Terapèutica Experimental, Facultat de Medicina i Ciències de la Salut, Universitat de Barcelona, Hospitalet de Llobregat, Spain; 3 Department of Clinical and Molecular Microbiology, University Hospital Centre Zagreb, Zagreb, Croatia; 4 Dept. of Biological Sciences, Munster Technological University, Cork, Ireland; 5 Health Services Laboratories, London, UK; 6 Radboud University Medical Centre, Nijmegen, The Netherlands; 7 Division of Human Bacterial diseases, Sciensano, Brussels, Belgium; 8 Laboratorie de Bactériologie, Plateforme de Biologie, Hôpital Cavale Blanche, Brest, France; 9 Division of Hygiene and Medical Microbiology, Medical University of Innsbruck, Innsbruck, Austria; 10 Servicio de Microbiología, Hospital Universitario Central de Asturias, Oviedo, Spain; 11 Servei de Microbiologia-CDB, Hospital Clínic de Barcelona-ISGlobal, Departament Fonaments Clínics, Facultat de Medicina i Ciències de la Salut, Universitat de Barcelona, Barcelona, Spain; 12 Servicio de Microbiología, Hospital Universitario A Coruña, Coruña, Spain; 13 Laboratorio de Microbiologia, Hospital de la Santa Creu i Sant Pau, Barcelona, Spain; 14 Institut de Microbiologie, Université de Lausanne, Lausanne, Switzerland; 15 Biomedical Center, Faculty of Medicine in Plzen, Plzen, Czech Republic; 16 Oslo University Hospital, Oslo, Norway; 17 Laboratorio de Microbiología, Hospital Regional de Málaga, Málaga, Spain; 18 Department of Medical Microbiology, MVZ Dr. Eberhard & Partner Dortmund, Dortmund, Germany

**Keywords:** Clinical microbiology, Infectious-disease diagnostics

## Abstract

The ability of MALDI-TOF for the identification of nontuberculous mycobacteria (NTM) has improved recently thanks to updated databases and optimized protein extraction procedures. Few multicentre studies on the reproducibility of MALDI-TOF have been performed so far, none on mycobacteria. The aim of this study was to evaluate the reproducibility of MALDI-TOF for the identification of NTM in 15 laboratories in 9 European countries. A total of 98 NTM clinical isolates were grown on Löwenstein-Jensen. Biomass was collected in tubes with water and ethanol, anonymized and sent out to the 15 participating laboratories. Isolates were identified using MALDI Biotyper (Bruker Daltonics). Up to 1330 MALDI-TOF identifications were collected in the study. A score ≥ 1.6 was obtained for 100% of isolates in 5 laboratories (68.2–98.6% in the other). Species-level identification provided by MALDI-TOF was 100% correct in 8 centres and 100% correct to complex-level in 12 laboratories. In most cases, the misidentifications obtained were associated with closely related species. The variability observed for a few isolates could be due to variations in the protein extraction procedure or to MALDI-TOF system status in each centre. In conclusion, MALDI-TOF showed to be a highly reproducible method and suitable for its implementation for NTM identification.

## Introduction

All species from the *Mycobacterium* genus except for *Mycobacterium tuberculosis* complex and *Mycobacterium leprae* are commonly known as non-tuberculous mycobacteria (NTM)^1^. This is an ever-growing group of bacteria (https://lpsn.dsmz.de/genus/mycobacterium) widely distributed in the environment^2^. The recommendation of the American Thoracic Society and Infectious Diseases Society of America (ATS/IDSA) and the British Thoracic Society is to provide species-level identification of NTM isolates from clinical origin in order to elucidate their clinical significance^3,4^.

In recent years, the identification of NTM in clinical laboratories has improved by the application of matrix-assisted laser desorption/ionization time-of-flight mass spectrometry (MALDI-TOF MS). On the one hand, improved commercial databases have increased the number of species that can be reliably identified by this system^5,6^. Moreover, in MALDI Biotyper system (Bruker Daltonics) the log(score) values ≥ 1.8 and ≥ 1.6 have been accepted for high-confidence and low-confidence identification of mycobacteria isolates, respectively, in order to increase their identification rate without loss of diagnostic accuracy^5^. Furthermore, several protein extraction procedures have been tested in order to efficiently break the cell wall of mycobacteria and extract their proteins^7–10^. This has been achieved by the use of chemical reagents (acetonitrile and formic acid) and mechanical disruption applying silica beads and vortexing, bead beating or, in some cases, by sonication^11–13^. Thanks to the previous procedures, now it is possible to obtain a rapid and reliable identification of NTM by MALDI-TOF MS.

There are few studies that have addressed the inter-laboratory reproducibility of MALDI-TOF MS so far, none on mycobacteria. Mainly they have focused on other bacterial species such as *Staphylococcus aureus*^14^, *Escherichia coli*^15^, nonfermenting bacteria^16^, anaerobic bacteria^17^ and mold^18^. They concluded that MALDI-TOF MS is a highly reproducible method between different laboratories, and attribute minor errors to the instruments or databases used.

The aim of this study was to evaluate the reproducibility of MALDI-TOF MS and performance of sonication procedure for identification of NTM in different laboratories from 9 European Economic Area (EEA) countries.

## Results

**Figure 1:**
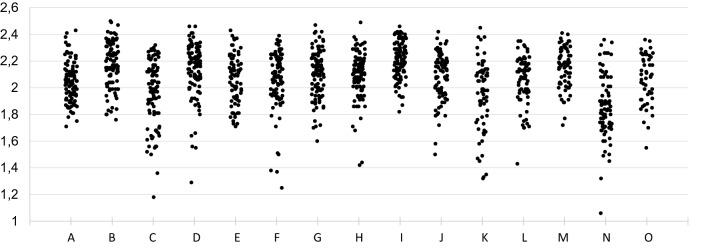
Distribution of isolates by centres and MALDI-TOF MS log(score).

A total of nine participating centres analysed all 98 mycobacterial isolates. In the other 6 laboratories, a lower number of isolates was analysed due to loss of samples during transport or during processing (Table 1). This resulted in a total of 1330 MALDI-TOF MS identifications performed. Globally, a log(score) ≥ 1.60 was obtained for 100% of the isolates in 5 laboratories, and between 68.2 and 98.6% of the cases in the other 10 centres (Table 1). The identification obtained by MALDI-TOF MS was 100% correct to complex-level in 12 laboratories (97.4–99.0% in the other 3 centres). Regarding species-level accuracy, identification was 100% correct in 8 participating centres (96.1–99.0% in the other 7 laboratories). Twelve (80.0%) centres reached a mean log(score) ≥ 2.0 in their analysis. Considering all 1330 identifications, 1243 (93.5%) obtained log(score) above 1.60. From these isolates with an accurate identification, 1239 (99.7%) were correct at complex-level and 1232 (99.1%) at species-level. Among the nine centres with all isolates analysed, Fleiss Kappa’s index (k = 0.917, *p* < 0.0001, confidence interval [CI] 95% 0.908–0.927) showed a highly accurate agreement between participants. Distribution of isolates by centres and according to the MALDI-TOF MS score is shown in Fig. 1.

**Table 1:** Identification results for all participating centres.

Centre	Isolates analysed	Log(score) ≥ 1.60 (%)	Complex-level correct (%)	Species-level correct (%)	Mean log(score)
A	98	100	100	100	2.04
B	98	100	100	100	2.17
C	98	87.8	100	100	1.96
D^a^	98	96.9	100	98.9	2.14
E	98	78.6	97.4	96.1	2.06
F	98	94.9	100	98.9	2.07
G	98	100	99	99	2.10
H	98	98	100	100	2.09
I	98	100	100	100	2.21
J	86	93	100	98.7	2.09
K	85	68.2	98.3	96.5	1.95
L	73	98.6	100	97.2	2.06
M	73	100	100	100	2.16
N	81	88.9	100	100	1.86
O	50	98	100	100	2.06
Total	1330	93.5	99.7	99.1	2.07

^a^MycoEX extraction procedure performed.

**Figure 2:**
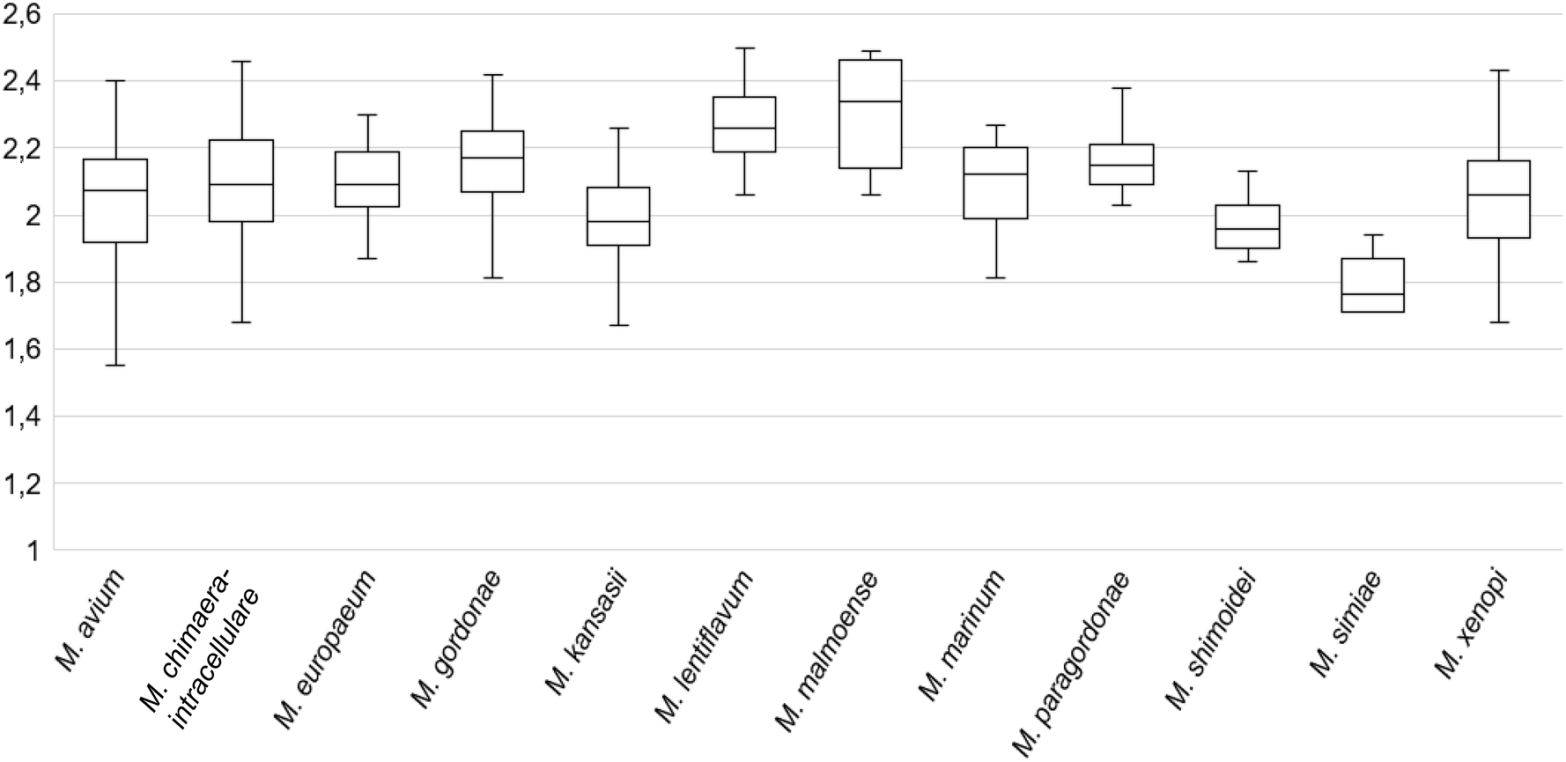
Log(score) obtained in all centres for slow growing species.

Among slow growing mycobacteria species, *Mycobacterium simiae* obtained the lowest log(score): mean of 1.76 (range 1.29–1.94), as observed in Fig. 2. The remaining species of this group obtained their identification with log(score) close to 2.0, with *Mycobacterium lentiflavum* (mean 2.25; range 1.87–2.50) and *Mycobacterium malmoense* (mean 2.27; range 1.47–2.49) yielding the highest log(score). Among isolates with log(score) < 1.60 or no peaks (87/1330; 6.5%), 68.1% were slow growing species.

**Figure 3:**
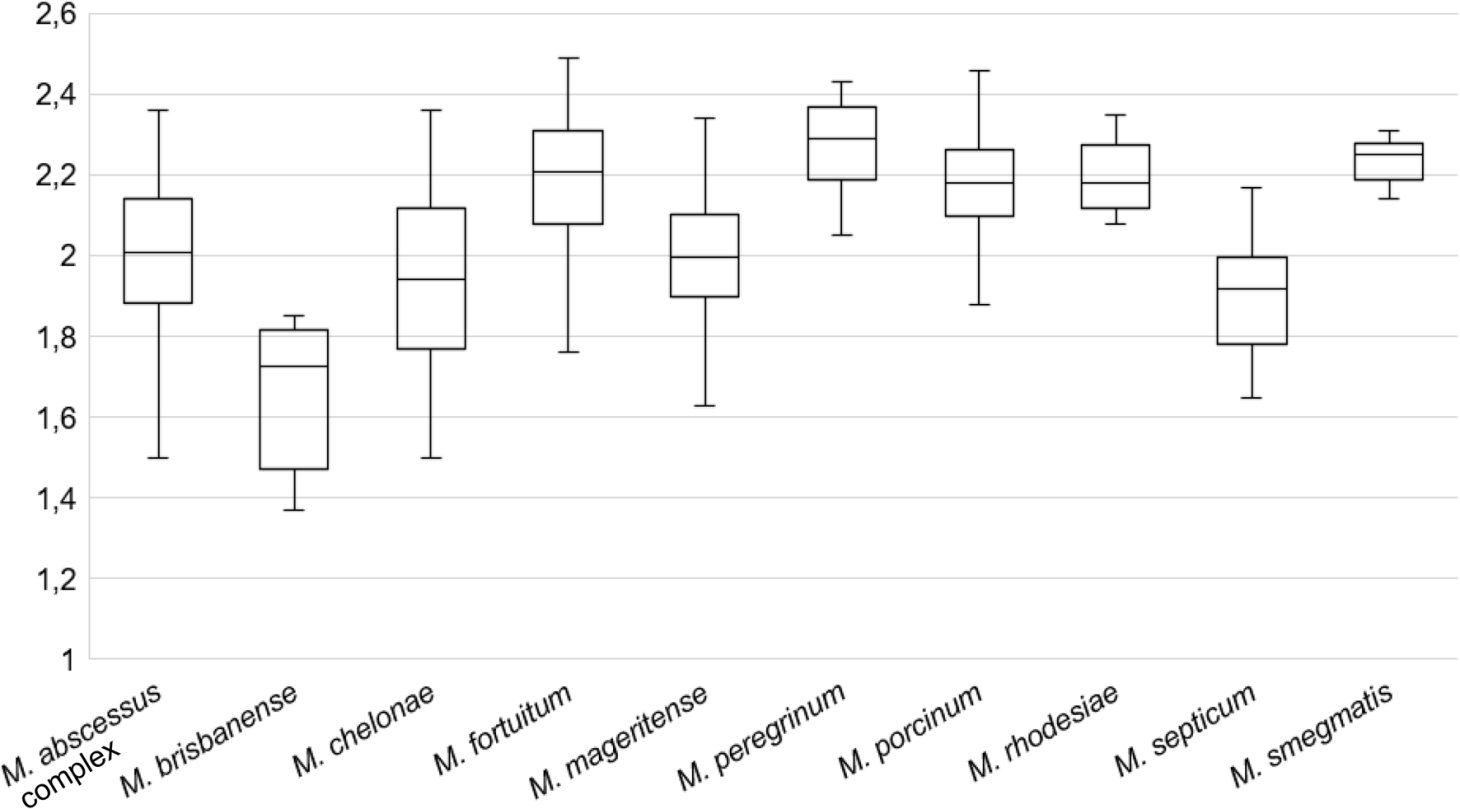
Log(score) obtained in all centres for rapid growing species.

Regarding rapid growing species, *Mycobacterium brisbanense* obtained log(score) lower than 1.80 (mean 1.66; range 1.37–1.85) in most of participating centres, as observed in Fig. 3. The species with higher log(score) were those belonging to *Mycobacterium fortuitum* complex (*M. fortuitum*, *Mycobacterium peregrinum* and *Mycobacterium porcinum*; mean 2.17; range 1.36–2.49), *Mycobacterium rhodesiae* (mean 2.19; range 2.08–2.35) and *Mycobacterium smegmatis* (mean 2.22; range 2.03–2.31).

According to their Kappa indexes, 21 out of the 22 species analysed obtained a high level of accuracy among laboratories, with a total concordance for *M. lentiflavum*, *M. malmoense*, *Mycobacterium shimoidei*, *Mycobacterium mageritense*, *M. peregrinum*, *M. rhodesiae* and *M. smegmatis* (Table 2). The species with lowest Kappa value was *M. simiae* (K = 0.748). Globally, 13 isolates obtained a misidentification in at least one centre. Among these, eight isolates were misidentified in all three replicated spots, and five obtained a misidentification in one of the three replicated spots (Table 3). Nine of the 13 misidentifications were related with rapid-growing mycobacteria: these involved close related species from *Mycobacterium chelonae-abscessus* complex and *M. fortuitum* complex. The other four strains were slow-growing mycobacteria, specifically from *Mycobacterium avium* complex and the species *Mycobacterium gordonae*, *Mycobacterium kansasii* and *Mycobacterium europaeum*. Regarding log(score) cut-offs, eight strains involved MALDI-TOF MS log(score) in high confidence category (score ≥ 1.80), and two of them above 2.00.

**Table 2:** Mycobacterial species included in the study and Kappa index obtained.

Slow growing species			Rapid growing species		
Species	N	Kappa index	Species	N	Kappa index
*M. avium*	8	0.970	*M. abscessus* complex	13	0.968
*M. chimaera-intracellulare*	11	0.877	*M. brisbanense*	1	0.874
*M. europaeum*	3	0.872	*M. chelonae*	4	0.906
*M. gordonae*	12	0.942	*M. fortuitum*	11	0.977
*M. kansasii*	7	0.983	*M. mageritense*	3	1.000
*M. lentiflavum*	6	1.000	*M. peregrinum*	2	1.000
*M. malmoense*	1	1.000	*M. porcinum*	4	0.970
*M. marinum*	1	0.874	*M. rhodesiae*	1	1.000
*M. paragordonae*	2	0.940	*M. septicum*	1	0.874
*M. shimoidei*	2	1.000	*M. smegmatis*	1	1.000
*M. simiae*	1	0.748			
*M. xenopi*	3	0.960

**Table 3:** Isolates that obtained some misidentification.

Strain number	Species	Centres with misidentification reported	Species identified	Number of replicated spots misidentified	Log(score)
4	*M. chelonae*	1	*M. salmoniphilum*	3/3	1.85–2.08
5	*M. paragordonae*	1	*M. gordonae*	3/3	1.94–2.09
6	*M. abscessus* complex	1	*M. fortuitum*	3/3	1.76–1.80
32	*M. chimaera-intracellulare*	1	*M. marseillense*	1/3	1.84
42	*M. chelonae*	2	*M. immunogenum*	3/3	1.67–1.82
		1	*M. salmoniphilum*	1/3	1.96
45	*M. abscessus* complex	1	*M. kansasii*	3/3	1.44–1.60
56	*M. abscessus* complex	1	*M. fortuitum*	1/3	1.91
59	*M. abscessus* complex	1	*M. immunogenum*	3/3	1.45–1.66
		1	Mixed species	3/3	
64	*M. septicum*	1	*M. peregrinum*	3/3	1.62–1.70
		1	*M. neworleansense*	1/3	1.71
67	*M. kansasii*	2	*M. gastri*	1/3	1.75–1.95
74	*M. europaeum*	1	*M. avium*	3/3	1.66–1.71
94	*M. porcinum*	1	*M. peregrinum*	1/3	1.80
99	*M. porcinum*	1	*M. peregrinum*	1/3	1.88
		1	*M. neworleansense*	1/3	1.97

## Discussion

Although MALDI-TOF MS has become a reference method for the identification of mycobacteria species in clinical microbiology laboratories, few studies have evaluated its inter-laboratory reproducibility so far and none of them has focussed on mycobacteria. This study collected identification data from 15 different laboratories that blindly analysed up to 98 mycobacterial strains, resulting in a total of 1330 MALDI-TOF MS identifications. As a result, more than 99% of isolates that obtained a satisfactory log(score) ≥ 1.60 were accurately identified at the species level. Besides, most of the isolates analysed in each participating centre reached a log(score) higher than 1.60 by MALDI-TOF MS. In fact, up to five of the participating laboratories obtained a log(score) above 1.60 in all isolates they analysed. The achievement of suitable MALDI-TOF MS log(score) in mycobacteria has improved over the last years thanks to optimized protein extraction protocols^11^ and the updating of the commercial databases, providing reliable identification that can be trusted by clinicians.

In this study, the sonication procedure was evaluated in 14 centres, leaving only one laboratory—proficient in the use of MALDI-TOF MS for the identification of mycobacteria isolates—that applied MycoEX protocol. The identification provided by this laboratory showed no important differences in comparison with other participants, who applied the sonication method (Table 1). Moreover, the model and the technical specifications of the mass spectrometer for spectra acquisition were common in all participating laboratories. In addition, all centres obtained mycobacterial isolates from the same culture, without need of growing them in each laboratory. All of this led to a few possible variables that could have interfered in the results obtained between different centres: (1) transportation and storage time of samples; (2) the technical experience of the personnel performing the protein extraction protocol^19^, although all participants had previous experience with MALDI-TOF MS; (3) the calibration and maintenance status of mass spectrometer in each participating laboratory.

Regarding differences between mycobacterial species, the score values observed could have been greatly influenced by the number of isolates included for each species and their representation in database. Thus, species with lower log(score) were *M. simiae*, *M. brisbanense* and *Mycobacterium septicum*, for which one isolate was included (Table 2). Although *M. simiae* and *M. septicum* are well represented in MALDI-TOF MS database, it is possible that the isolates included in this study had different intrinsic characteristics than those in the database. In the case of *M. brisbanense*, only two spectra are included in current database. Conversely, species with higher log(score) and identification rate were *M. malmoense* (n = 1) and *M. lentiflavum* (n = 6) for slow-growing mycobacteria and *M. fortuitum* complex (n = 11), *M. rhodesiae* (n = 1) and *M. smegmatis* (n = 1) for rapid-growing. With the exception of *M. rhodesiae*, all previous species are widely represented in database used.

Mycobacterial misidentifications by MALDI-TOF MS have been reported in literature, even with high log(score)^20^. Fortunately, this is not a common phenomenon. Misidentifications usually arise due to some species being very closely related or pertaining to the same mycobacterial complex. In this study, three replicates were used for analysis of each isolate. Although more than 99% of isolates have been correctly identified at species-level, a total of 13 different isolates obtained at least one misidentification in some replicates by one of the participating centres (Table 3). Some of the misidentifications reported involved only one of these three replicates, so they could probably have been caused by technical issues during spectra acquisition in this spot. When three replicates are used, same identification in two of them may be considered as reliable identification. According to this statement, only 10 (0.008%) isolates from 1243 analyses were definitely misidentified (Table 3).

Most of the misidentifications obtained were reported as other species of the same group, except strain number 56 (*M. abscessus* complex), which was identified as *M. fortuitum* in one spot (score = 1.91). Regarding misidentifications involving all three replicates, most of them were from rapid-growing species, such as *M. chelonae* and *M. abscessus*. Although these two species were not confused, some laboratories identified one of these isolates as *Mycobacterium salmoniphilum* and *Mycobacterium immunogenum*, two species closely related to *M. chelonae*^21,22^. The most controversial misidentifications were the following: (1) one centre that identified a *M. abscessus* isolate as *M. kansasii*, even though it obtained a low log(score) (1.60); (2) a *M. abscessus* with mixed species identified in three replicates, maybe due to contamination of tubes or MALDI plate spots during preparation of sample; and (3) one *M. europaeum* identified as *M. avium* in one centre. For the lattermost misidentification, it could not be definitively determined if it was a MALDI-TOF MS misidentification or due to contamination. In all cases, sample interchange was ruled out.

In summary, MALDI-TOF MS demonstrated an extraordinary inter-laboratory reliability for the identification of NTMs, reaching a high concordance among identifications obtained in participating centres. Besides, sonication of the bacterial pellet has shown to be a good alternative to the MycoEX procedure^20^. Misidentifications were scarce and they were considered as minor errors since the identifications provided by the MALDI-TOF MS corresponded to species from the same complex. Although little improvements can still be made in databases, MALDI-TOF MS showed to be a highly reproducible technique, and therefore, it could be globally implemented for rapid, accurate and first line identification method for mycobacteria in clinical microbiology laboratories.

## Methods

### Mycobacterial strains and participating centres

A total of 98 NTM clinical isolates were analysed in 15 laboratories from 9 European Economic Area (EEA) countries (Austria, Belgium, Croatia, France, Germany, Ireland, Norway, Spain and United Kingdom). The strains were recovered from frozen stocks and grown on Löwenstein-Jensen (Becton Dickinson, Franklin Lakes, NJ) in the Department of Clinical Microbiology and Infectious Diseases of the Hospital General Universitario Gregorio Marañón (Madrid, Spain). They encompassed 22 different mycobacterial species (Table 2), previously identified by sequencing of *16S rRNA* and/or *hsp65* genes. A 10 µl loop was used to collect biomass from colonies in tubes with 300 µl water and 900 µl ethanol, sent out to the participating laboratories and blindly analysed.

### MALDI-TOF MS protein extraction protocol

In 14 centres, the protein extraction procedure was performed by sonication as previously described^11,23^ and the remaining laboratory applied Myco-EX protocol recommended by the manufacturer. Upon arrival in the laboratory, the tubes with the samples were centrifuged 5 min at 14,000 rpm and the pellet was resuspended in 300 µl of high-performance liquid chromatography (HPLC) or sterile Milli-Q water, after which they were heat inactivated at 95 °C for 30 min. After the addition of 900 µl of ethanol, the tubes were centrifuged for 2 min at 14,000 rpm and the supernatant was discarded. The pellet was dried for a few minutes at room temperature. Then, a spatula tip with 0.5 mm diameter glass or zirconia/silica beads (BioSpec products, USA) and 50 µl of acetonitrile were added. The tubes were vortexed for 10 s. In 14 laboratories, an additional step of 15 min sonication was performed. After this, 50 µl of formic acid were added and the tubes were vortexed again for 10 s. Finally, the samples were centrifuged for 2 min at 14,000 rpm and 1 µl of the supernatant was deposited by triplicate onto the MALDI plate (Bruker Daltonics, Bremen, Germany). After drying, 1 µl of HCCA (α-cyano-4-hydroxycinnamic acid) matrix was added to the spots and dried at room temperature.

### MALDI-TOF MS analysis

In all participating centres, the MALDI-TOF system used was a Biotyper microflex LT (Bruker Daltonics). The software used was FlexControl v3.0 with the Mycobacteria Library v4.0. The spectra were obtained in the positive linear mode, over a mass/charge (*m/z*) ratio of 2000–20,000 Da and the accelerating voltage was 20 kV. The samples were measured in automatic mode using a nitrogen laser at 40 shots per second, with a total of 240 laser shots collected per spot. The log(score) cut-offs used were those recommended by the manufacturer: < 1.60 as not reliable identification, 1.60–1.79 as low confidence identification and a log(score) ≥ 1.80 as high confidence identification.

### Statistical analysis

Fleiss Kappa index was applied to measure the concordance of the results from different centres regarding the reference method (low < 0.40; moderate 0.41–0.60; accurate 0.61–0.80; very accurate > 0.81). In order to evaluate the same number of isolates, only those centres with all isolates analysed were selected for statistics using SPSS 28.0 software (IBM, NY, USA).
